# Neem Oil and Crop Protection: From Now to the Future

**DOI:** 10.3389/fpls.2016.01494

**Published:** 2016-10-13

**Authors:** Estefânia V. R. Campos, Jhones L. de Oliveira, Mônica Pascoli, Renata de Lima, Leonardo F. Fraceto

**Affiliations:** ^1^Department of Environmental Engineering, São Paulo State UniversitySorocaba, Brazil; ^2^Department of Biochemistry, Institute of Biology, State University of CampinasCampinas, Brazil; ^3^Department of Biotechnology, University of SorocabaSorocaba, Brazil

**Keywords:** neem oil, nanoparticles, sustained release, sustainable agriculture

## Abstract

A major challenge of agriculture is to increase food production to meet the needs of the growing world population, without damaging the environment. In current agricultural practices, the control of pests is often accomplished by means of the excessive use of agrochemicals, which can result in environmental pollution and the development of resistant pests. In this context, biopesticides can offer a better alternative to synthetic pesticides, enabling safer control of pest populations. However, limitations of biopesticides, including short shelf life, photosensitivity, and volatilization, make it difficult to use them on a large scale. Here, we review the potential use of neem oil in crop protection, considering the gaps and obstacles associated with the development of sustainable agriculture in the not too distant future.

## Introduction

Attention is increasingly being paid to the use of natural compounds (such as essential oils) as a promising option to replace agrochemicals in agricultural pest control. These odoriferous substances are extracted from various aromatic plants, which are rich sources of biologically active secondary metabolites such as alkaloids, phenolics, and terpenoids (Esmaeili and Asgari, 2015), using extraction methods employing aqueous or organic solvents, or steam distillation. Their mechanisms of action can vary, especially when the effect is due to a combination of compounds (de Oliveira, 2011; Esmaeili and Asgari, 2015).

Neem oil is extracted from the neem tree, *Azadirachta indica* Juss., a member of the *Meliaceae* family that originates from the Indian subcontinent and is now valued worldwide as an important source of phytochemicals for use in human health and pest control. *Azadirachta* is a fast-growing small-to-medium sized evergreen tree, with wide and spreading branches. It can tolerate high temperatures as well as poor or degraded soil. The young leaves are reddish to purple, while the mature leaves are bright green, consisting of petiole, lamina, and the base that attaches the leaf to the stem and may bear two small lateral leaf-like structures known as stipules (Norten and Pütz, 1999; Forim et al., 2014).

**FIGURE 1:**
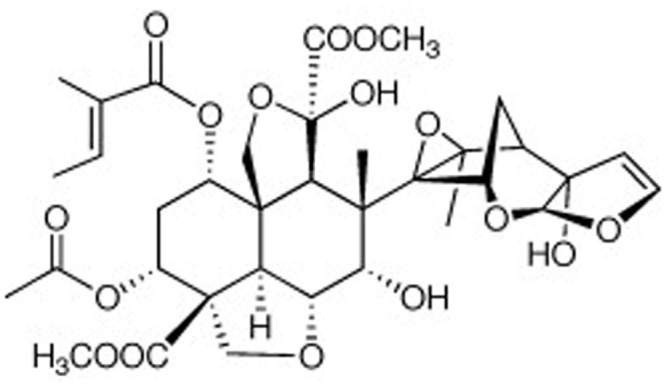
**Chemical structure of azadirachtin, the main component of neem oil**.

Neem oil contains at least 100 biologically active compounds. Among them, the major constituents are triterpenes known as limonoids, the most important being azadirachtin (**Figure 1**), which appears to cause 90% of the effect on most pests. The compound has a melting point of 160°C and molecular weight of 720 g/mol. Other components present include meliantriol, nimbin, nimbidin, nimbinin, nimbolides, fatty acids (oleic, stearic, and palmitic), and salannin. The main neem product is the oil extracted from the seeds by different techniques. The other parts of the neem tree contain less azadirachtin, but are also used for oil extraction (Nicoletti et al., 2012). It has been suggested that the content of azadirachtin in the seeds can be increased by artificial infection with arbuscular mycorrhiza (Venkateswarlu et al., 2008).

Among the botanical insecticides currently marketed, neem oil is one of the least toxic to humans and shows very low toxicity to beneficial organisms, so it is, therefore, very promising for the control of many pests. Target insect species include the following: *Anopheles stephensi* (Lucantoni et al., 2006), *A. culicifacies* (Chandramohan et al., 2016), *Ceraeochrysa claveri* (Scudeler et al., 2013, 2014; Scudeler and dos Santos, 2013), *Cnaphalocrocis medinalis* (Senthil Nathan et al., 2006), *Diaphorina citri* (Weathersbee and McKenzie, 2005), *Helicoverpa armigera* (Ahmad et al., 2015), *Mamestra brassicae* (Seljåsen and Meadow, 2006), *Nilaparvata lugens Stal* (Senthil-Nathan et al., 2009), *Pieris brassicae* (Hasan and Shafiq Ansari, 2011), and *Spodoptera frugiperda* (Tavares et al., 2010). Arachnid targets include *Hyalomma anatolicum excavatum* (Abdel-Shafy and Zayed, 2002) and *Sarcoptes scabie* var. cuniculi larvae (Xu et al., 2010).

The oil is considered a contact insecticide, presenting systemic and translaminar activity (Cox, 2002). It has a broad spectrum of action, inhibiting feeding, affecting hormone function in juvenile stages, reducing ecdysone, deregulating growth, altering development and reproduction, suppressing fertility, sterilizing, repelling oviposition, and disrupting molting processes (Brahmachari, 2004). Little is known about the mode of action of azadirachtin as a feeding inhibitor, although it is possible that it stimulates cells involved in feeding inhibition, causing weakness and pest death (Brahmachari, 2004).

Azadirachtin, salannin, and other limonoids present in neem oil inhibit ecdysone 20-monooxygenase, the enzyme responsible for catalyzing the final step in conversion of ecdysone to the active hormone, 20-hydroxyecdysone, which controls the insect metamorphosis process. However, these effects are probably secondary to the action of azadirachtin in blocking microtubule formation in actively dividing cells (Morgan, 2009). Moreover, azadirachtin can inhibit the release of prothoracicotropic hormone and allatotropins from the brain-corpus cardiacum complex, resulting in problems of fertility and fecundity (Mulla and Su, 1999). Meliantriol and salannin also act to inhibit the feeding of insects, while nimbin and nimbidin mainly present antiviral activity (EMBRAPA, 2008).

Azadirachtin can also interfere in mitosis, in the same way as colchicine, and has direct histopathological effects on insect gut epithelial cells, muscles, and fatty tissues, resulting in restricted movement and decreased flight activity (Wilps et al., 1992; Mordue (Luntz) and Blackwell, 1993; Qiao et al., 2014).

Several studies have described the action of neem oil in specific groups of insects. Among the major insect groups, neem oil has shown action against (i) Lepidoptera: antifeeding effect and increased larvae mortality (Mancebo et al., 2002; Michereff-Filho et al., 2008; Tavares et al., 2010); (ii) Hemiptera: early death of nymphs in due to inhibition of development and ecdysis defects (Weathersbee and McKenzie, 2005; Senthil Nathan et al., 2006; Formentini et al., 2016); (iii) Hymenoptera: food intake decrease, reduced larval and pupal development, larvae death during the molting process (Li et al., 2003); (iv) Neuroptera: severe damage in the midgut cells of larvae, injury and cell death during the replacement of midgut epithelium, and changes in cocoons, with increased porosity and decreased wall thickness affecting pupation (Scudeler et al., 2013, 2014; Scudeler and dos Santos, 2013). In another class, the Arachnida, exposure of the Ixodidae group to neem oil decreased egg hatching and caused malformation, deformities, and death of larvae and adults (Abdel-Shafy and Zayed, 2002).

## Neem Applications

For centuries, neem has been used in folk medicine for the treatment of conditions such as malaria, ulcers, cardiovascular disease, and skin problems. Despite the limited existence of clinical trials to support therapeutic claims, the use of neem has expanded over time, and it is an important component of Ayurvedic medicine (medical knowledge developed in India about 7000 years ago; Girish and Shankara Bhat, 2008; Ogbuewu et al., 2011).

**FIGURE 2:**
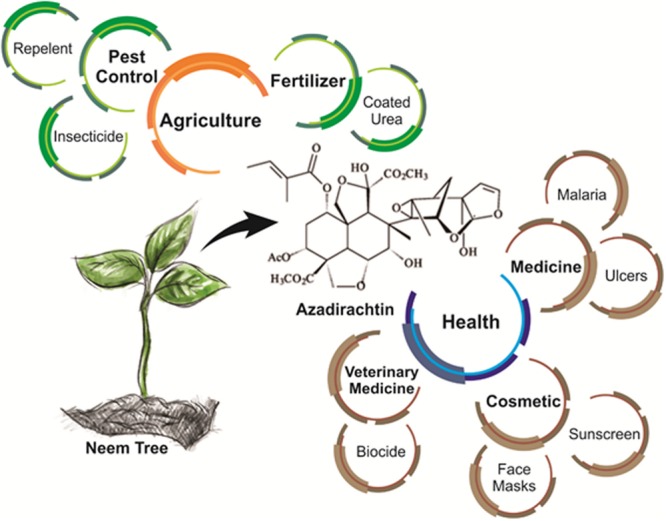
**Potential applications of azadirachtin in different areas**.

In addition to its medical applications, neem has aroused interest in many other areas (**Figure 2**). In the cosmetics and hygiene sector, neem is used in the composition of face masks, lotions, sunscreens, soaps, and toothpastes (Mathur and Kachhwaha, 2015). Products derived from neem can contribute to sustainable development and the resolution of pest control problems in agriculture (Lokanadhan et al., 2012). These products benefit from the natural properties of neem as a powerful insect growth regulator (IGR) that also affects many other organisms (such as nematodes and fungi) and can act as a plant fertilizer (Brahmachari, 2004).

The use of neem in agriculture is not a new practice. In India, the traditional farming system employed neem extracts for pest management and to supply nutrients to plants (Mossini and Kemmelmeier, 2005; Sujarwo et al., 2016). Scientific research has shown that neem is safe for workers, with no handling risks, and can be used throughout the entire crop production cycle (Boeke et al., 2004).

Neem has proven use as a fertilizer, with the organic and inorganic compounds present in the plant material acting to improve soil quality and enhance the quality and quantity of crops. The waste remaining after extraction of the oil from neem seeds (neem seed cake) can be used as a biofertilizer, providing the macronutrients essential for plant growth (Ramachandran et al., 2007; Lokanadhan et al., 2012).

Nitrogen is one of the main nutrients required by plants for their development, and urea is the main source of nitrogen fertilizer used worldwide to supply the nitrogen demand of crops. The control of urea hydrolysis and nitrification is one of the principal strategies employed to avoid nitrogen losses in agriculture (Ni et al., 2014). Neem has demonstrated activity as a nitrification inhibitor, helping to slow the bacterial activity that is responsible for denitrification, hence decreasing the loss of urea from the soil (Musalia et al., 2000; Mohanty et al., 2008).

Due to their compositional complexity, neem-based products can act as antifeedants, growth regulators, sterilants, anti-oviposition agents, and repellents (Gonzalez-Coloma et al., 2013). Other factors that have stimulated the use of neem-based products for pest control in agriculture are ecological and toxicological aspects (low toxicity to non-target organisms), as well as economic aspects (small amounts of the product can provide effective pest control; Ogbuewu et al., 2011).

These features of neem support its contribution to organic agricultural production systems that are more sustainable and do not generate chemical residues (plants and crops are grown without the use of any agrochemicals). This method also helps to maintain soil productivity, ensuring longer production times. Organic agriculture can be a viable alternative production method for farmers, but there are numerous challenges to be overcome. A key to success is to be open to new approaches, and in this respect neem products can effectively contribute to organic agriculture, being used as organic pesticides and as soil fertilizers. In addition, growing concerns about conventional agriculture and the demand for products that do not generate waste justify increased adoption of the use of biopesticides by farmers, which contributes to the growth of organic agriculture (Dubey et al., 2010; Seufert et al., 2012; Gahukar, 2014).

## Commercial Products Derived From Neem (*Azadirachta indica*)

Neem has acquired commercial recognition due to its various beneficial properties, which have been extensively investigated over time. Compared to conventional chemicals, which are generally persistent in the environment and highly toxic, botanical pesticides are biodegradable and leave no harmful residues. Most botanical pesticides are non-phytotoxic and are also more selective toward the target pest. In terms of commercial applications, biopesticides can provide substantial economic advantages, since the infrastructure required is inexpensive, compared to conventional pesticides (Pant et al., 2016).

This has resulted in the publication of numerous scientific research articles and books, as well as the organization of international conferences to discuss the benefits of the plant (Girish and Shankara Bhat, 2008).

Several patents related to processes and products based on neem have been deposited in the United States, India, Japan, Australia, and elsewhere. Many of the products derived from neem are manufactured by crushing the seeds and other plant parts, followed by the use of solvents to extract the active ingredients possessing pesticide activity. The different methods and techniques employed to obtain neem products can result in different concentrations of the active compounds, as well as different biological effectiveness (Roychoudhury, 2016). **Table 1** lists some of the main commercial products based on neem.

**Table 1:** Neem applications and commercial products available worldwide.

Application	Product	Manufacturer
Fertilizer	Ozoneem Cake^®^	Ozone Biotech (India)
	Plan “B” Organics – Neem Cake^®^	Plan “B” Organics (USA)
	Fortuneem Cake^®^	Fortune Biotech (USA)
	Bio Neem Oil Foliar^®^	FUSA – Fertilizers of the USA
	Neem Cake^®^	Unibell Corporation (Russia)
	Ozoneem Coat^®^	Ozone Biotech (India)
	Parker Neem Coat^®^	Parker Neem (India)
	Neem Urea Guard^®^	Neemex (India)
	Fortuneem Coat^®^	Fortune Biotech (USA)
	*Azadirachtin-based products*	
Agrochemical	AZA-Direct^®^	Gowan Company (USA)
	Neemix 4.5^®^	Certis (USA)
	Fortune Aza 3% EC^®^	Fortune Biotech (USA)
	Azamax^®^	UPL Ltda. (Brazil)
	Neemazal Technical^®^	E.I.D. Parry Ltd. (India)
	Ecosense^®^	Agro Logistic Systems Inc. (USA)
	Safer Brand 3 in 1	Woodstream Corp. (Canada)
	Garden Spray^®^
	Azatin XL^®^	OHP Inc. (USA)
	Azact CE^®^	EPP Ltda. (Brazil)
	*Neem oil*	
	Triact 70 EC^®^	Certis Company (USA)
	BioNeem^®^	Woodstream Corporation (USA)
	Shubhdeep Neem Oil^®^	King Agro Food (India)
	DalNeem^®^	Dalquim Ltda. (Brazil)
	OzoNeem Oil^®^	Ozone Biotech (India)
	NeemDrop^®^	Neem India Products Ltd. (India)

Despite its many promising properties, there are limitations that hinder effective large-scale use of neem. These impediments must be overcome and many uncertainties clarified so that the full potential of neem can be exploited. One of the main problems facing the commercial development of neem is a lack of industrial interest, largely due to the difficulty of patenting natural products, as well as a shortage of scientific evidence to support claims regarding the benefits of these substances. As a results, the products are not widely publicized in the farming community and elsewhere (Pant et al., 2016).

Disadvantages of neem are its low stability under field conditions, due mainly to a high rate of photodegradation, as well as a short residence time and slow killing rates, compared to conventional pesticides (Isman, 2006; de Oliveira et al., 2014; Miresmailli and Isman, 2014). Genetic factors are mainly responsible for determining the chemical composition of neem oil. However, environmental factors and the type of extraction method can lead to significant differences in composition. As a result, there is no standard active ingredient in the composition of this botanical insecticide, which limits its application in the control of agricultural pests (Ghosh et al., 2012; Tangtrakulwanich and Reddy, 2014; Siegwart et al., 2015).

Neem oil contains a group of active ingredients with different chemical characteristics. It was therefore believed that the development of insect resistance would be virtually impossible. However, as studies have progressed, it has been observed that due to the low residual power of botanical insecticides, multiple applications are required in order to control pests, which can increase selection pressure on the pest population, possibly leading to resistance (Ghosh et al., 2012; Tangtrakulwanich and Reddy, 2014; Siegwart et al., 2015).

Currently, most of the botanical insecticides that are being studied and that are effective against many pests are those with feeding deterrent action, so their indiscriminate use could result in the development of resistance (Tangtrakulwanich and Reddy, 2014; Mpumi et al., 2016). Feng and Isman (1995) evaluated the behavior of two lines of *Myzus persicae*, which were exposed to pure azadirachtin or to refined neem seed extract at the same concentration as azadirachtin. It was found that after forty generations, the line treated with azadirachtin had developed ninefold greater resistance to azadirachtin, compared to a control line, whereas the line treated with the extract did not show resistance.

## Future Trends

Biological control is defined as the action of natural enemies on a population of pests in order to keep it at a population density that does not cause economic damage to crops (Pal and McSpadden Gardener, 2006). Natural enemies have been known since the third century BC, when the Chinese used predatory ants for pest control in citrus. However, after 1939, with the synthesis of the chlorinated pesticide dichlorodiphenyltrichloroethane (DDT) and organophosphorus pesticides, research on synthetic chemical pesticides and their use increased greatly, while the opposite occurred with biological control methods (Doutt, 1964; Niu et al., 2014). Currently, with the emergence of the concept of Integrated Pest Management (IPM), there is a resurgence of research with emphasis on biological control techniques. Such systems seek to harmoniously integrate various forms of control, with emphasis on biological control, in order to gain economic, social, and environmental improvements (Kogan, 1998; Ehler, 2006; EPA, 2016).

The biological control of insects and mites in agriculture can be achieved using small wasps or flies, known as parasitoids, which parasitize eggs, small caterpillars, and even adults. It can also be performed using predators such as ladybugs, bugs, predatory mites, and spiders, as well as parasitism by entomopathogenic microorganisms including fungi, bacteria, and viruses (Landis et al., 2000; Ehler, 2006; Smith and Capinera, 2014). Although biological control will not control all pests all of the time, it is a key component of integrated pest management. The purpose of biological control is not to eradicate pests, but to keep them at tolerable levels at which they cause no appreciable harm (Orr and Lahiri, 2014).

There has recently been increased interest in the application of plant-based materials (botanical insecticides), such as neem oil, in pest control. Although these products are safer for the management of pests, compared to synthetic chemicals, their effects in IPM must be evaluated. Several studies have investigated the relationships between botanical insecticides and natural enemies of agricultural pests (Islam et al., 2011; Mamoon-ur-Rashid et al., 2011; Islam and Omar, 2012; Tunca et al., 2012; Usman et al., 2012). Sahayaraj et al. (2011) evaluated the use of different neem-based products in colonies of *Beauveria bassiana, Isaria fumosoroseus*, and *Lecanicillium lecanii*, and the results showed that these entomopathogenic fungi were compatible with most products tested. Raguraman and Kannan (2014) conducted a review in order to score the impact and safety of different botanical insecticides in the presence of parasitoids and predators (beneficial arthropods), with the aim of standardizing strategies and application methods to achieve better management of agricultural pests.

The integrated use of botanical insecticides associated with biological control (synergism) in IPM is becoming increasingly widespread in the farming and research communities. The advantage of this approach is that it offers the potential to control agricultural pests, without serious impacts on the environment, non-target organisms, and animal and human health.

Botanical insecticides must meet the same criteria as conventional insecticides. In other words, they must be selective for the target pest and provide sufficient residual activity to protect the plant during the period of vulnerability. Over the past decade, there has been a significant increase in the number of publications concerning the use of neem oil to control agricultural pests (Montes-Molina et al., 2008; War et al., 2012; da Costa et al., 2014; Gahukar, 2014; Rehman et al., 2014; Bakry et al., 2016). However, many studies have only involved testing at the laboratory level (*in vitro*), due to the instability of this substance under field conditions. From these studies, it is not possible to draw firm conclusions concerning the in vivo biological efficacy of the formulations, due to the effects of numerous environmental variables.

In order to overcome the above-mentioned limitations, nanotechnology has emerged as a novel tool to address the problems of agricultural sustainability and food security (Khot et al., 2012; Kah and Hofmann, 2014; Kookana et al., 2014; Kah, 2015; Kashyap et al., 2015; Fraceto et al., 2016). Many studies have shown that the encapsulation of agrochemicals in nanoparticulate systems can enhance the efficacy of the active ingredient, decrease toxicity toward the environment and humans, and reduce losses due to volatilization, leaching, and photobleaching (Kulkarni et al., 1999; Riyajan and Sakdapipanich, 2009; Devi and Maji, 2010; de Oliveira et al., 2014; Bakry et al., 2016; Giongo et al., 2016).

From the point of view of sustainable agriculture, nanotechnology can help in the development of environmentally friendly agricultural inputs, improving the safety and stability of active agents, enhancing their activity in pest control, and, consequently, increasing their acceptance by producers (Nair et al., 2010; Srilatha, 2011; Khot et al., 2012; Agrawal and Rathore, 2014; Ram et al., 2014). The use of nanoparticles provides an effective means of protecting neem oil against premature degradation, resulting in prolongation of its effect on the target pest. Sustained release of the active agent is achieved, and environmental damage is minimal because the polymers employed are biodegradable. Furthermore, the number of applications of neem oil can be reduced, bringing substantial economic benefits (Kulkarni et al., 1999; Isman et al., 2001; Isman, 2006; de Oliveira et al., 2014; Isman and Grieneisen, 2014; Miresmailli and Isman, 2014).

Although studies have demonstrated the beneficial effects of nanoencapsulation of neem oil, some issues need to be resolved so that the synergistic effect of nanoparticles associated with this botanical insecticide can significantly contribute to the control of insect pests. These issues include the need for: (a) regulation of the use of nanomaterials in agriculture; (b) nanoformulations that are easily scalable; (c) comparative studies employing neem formulations available commercially to prove the cost/benefit of nanoformulations; (d) detailed studies of the degradation and behavior of these nanopesticides in the environment; and (e) evaluation of toxicity toward non-target organisms (De Jong and Borm, 2008; Joint Research Centre, 2015; Servin and White, 2016).

Given the importance of neem oil and its worldwide use for combating numerous pests in different crops, the nanoencapsulation of this oil should enable the production of more stable formulations for the control of insects that damage crops, especially those that are essential for human consumption. In addition, the use of nanotechnology is an excellent way to combat the development of resistance in insects due to the indiscriminate use of neem oil.

## Author Contributions

EC, JdO, and MP wrote the manuscript. LF and RdL contributed to the discussion and revised the manuscript. All authors approved the final manuscript.

## Conflict of Interest Statement

The authors declare that the research was conducted in the absence of any commercial or financial relationships that could be construed as a potential conflict of interest.

## References

[B1] Abdel-Shafy S. Zayed A. A. (2002). In vitro acaricidal effect of plant extract of neem seed oil (*Azadirachta indica*) on egg, immature, and adult stages of *Hyalomma anatolicum* excavatum (Ixodoidea: Ixodidae). *Vet. Parasitol.* 106 89–96. 10.1016/S0304-4017(02)00023-7 11992715

[B2] Agrawal S. Rathore P. (2014). Nanotechnology pros and cons to agriculture: a review. *Int. J. Curr. Microbiol. Appl. Sci.* 3 43–55.

[B3] Ahmad S. Ansari M. S. Muslim M. (2015). Toxic effects of neem based insecticides on the fitness of *Helicoverpa armigera* (Hübner). *Crop Prot.* 68 72–78. 10.1016/j.cropro.2014.11.003

[B4] Bakry A. M. Abbas S. Ali B. Majeed H. Abouelwafa M. Y. Mousa A. (2016). Microencapsulation of oils: a comprehensive review of benefits, techniques, and applications: encapsulation of marine, vegetable, essential oils. *Compr. Rev. Food Sci. Food Saf.* 15 143–182. 10.1111/1541-4337.12179 33371581

[B5] Boeke S. J. Boersma M. G. Alink G. M. van Loon J. J. van Huis A. Dicke M. (2004). Safety evaluation of neem (*Azadirachta indica*) derived pesticides. *J. Ethnopharmacol.* 94 25–41. 10.1016/j.jep.2004.05.011 15261960

[B6] Brahmachari G. (2004). Neem–an omnipotent plant: a retrospection. *Chembiochem* 5 408–421. 10.1002/cbic.200300749 15185362

[B7] Chandramohan B. Murugan K. Madhiyazhagan P. Kovendan K. Kumar P. M. Panneerselvam C. (2016). Neem by-products in the fight against mosquito-borne diseases: biotoxicity of neem cake fractions towards the rural malaria vector *Anopheles culicifacies* (Diptera: Culicidae). *Asian Pac. J. Trop. Biomed.* 6 472–476. 10.1016/j.apjtb.2015.11.013

[B8] Cox C. (2002). Pyrethrins/pyrethrum insecticide factsheet. *J. Pestic. Reform* 22 14–20.

[B9] da Costa J. T. Forim M. R. Costa E. S. De Souza J. R. Mondego J. M. Boiça Junior A. L. (2014). Effects of different formulations of neem oil-based products on control *Zabrotes subfasciatus* (Boheman, 1833) (Coleoptera: Bruchidae) on beans. *J. Stored Prod. Res.* 56 49–53. 10.1016/j.jspr.2013.10.004

[B10] De Jong W. H. Borm P. J. (2008). Drug delivery and nanoparticles: applications and hazards. *Int. J. Nanomed.* 3 133–149. 10.2147/IJN.S596 PMC2527668 18686775

[B11] de Oliveira A. R. M. (2011). Análise enantiosseletiva de fármacos e metabólitos empregando eletroforese capilar. *Sci. Chromatogr.* 3 231–247.

[B12] de Oliveira J. L. Campos E. V. R. Bakshi M. Abhilash P. C. Fraceto L. F. (2014). Application of nanotechnology for the encapsulation of botanical insecticides for sustainable agriculture: prospects and promises. *Biotechnol. Adv.* 32 1550–1561. 10.1016/j.biotechadv.2014.10.010 25447424

[B13] Devi N. Maji T. K. (2010). Genipin crosslinked microcapsules of gelatin A and κ-carrageenan polyelectrolyte complex for encapsulation of Neem (*Azadirachta Indica* A. Juss.) seed oil. *Polym. Bull.* 65 347–362. 10.1007/s00289-010-0246-5

[B14] Doutt R. L. (1964). “The historical development of biological control,” in *Biological Control of Insect Pests and Weeds* ed. DeBach P. (New York, NY: Reinhold Publishing Corporation).

[B15] Dubey N. K. Shukla R. Kumar A. Singh P. Prakash B. (2010). Prospects of botanical pesticides in sustainable agriculture. *Curr. Sci.* 98 479–480.

[B16] Ehler L. E. (2006). Integrated pest management (IPM): definition, historical development and implementation, and the other IPM. *Pest Manag. Sci.* 62 787–789. 10.1002/ps.1247 16786545

[B17] EMBRAPA (2008). *A Cultura do Nim/Embrapa Florestas* 1st Edn. Brasília: Embrapa Informação Tecnológica.

[B18] EPA (2016). *Integrated Pest Management (IPM) Principles.* Available at: https://www.epa.gov/safepestcontrol/integrated-pest-management-ipm-principles (accessed August 26, 2016).

[B19] Esmaeili A. Asgari A. (2015). In vitro release and biological activities of *Carum copticum* essential oil (CEO) loaded chitosan nanoparticles. *Int. J. Biol. Macromol.* 81 283–290. 10.1016/j.ijbiomac.2015.08.010 26257380

[B20] Feng R. Isman M. B. (1995). Selection for resistance to azadirachtin in the green peach aphid, *Myzus persicae*. *Experientia* 51 831–833. 10.1007/BF01922438

[B21] Forim M. R. Fernandes D. S. M. F. Fernandes J. B. Vieira P. C. (2014). *Processo de Obtenção de Nanopartículas Biopoliméricas Contendo Óleo e Extratos de Azadirachta Indica a. Juss (neem), Nanopartículas Biopoliméricas e Micropartículas em pó.* Available at: http://www.google.co.ve/patents/WO2014113860A1 [accessed April 3, 2016].

[B22] Formentini M. A. Alves L. F. A. Schapovaloff M. E. (2016). Insecticidal activity of neem oil against *Gyropsylla spegazziniana* (Hemiptera: Psyllidae) nymphs on Paraguay tea seedlings. *Braz. J. Biol.* 10.1590/1519-6984.04915 [Epub ahead of print]. 27143053

[B23] Fraceto L. F. Grillo R. de Medeiros G. A. Scognamiglio V. Rea G. Bartolucci C. (2016). Nanotechnology in agriculture: which innovation potential does it have? *Front. Environ. Sci.* 4:20 10.3389/fenvs.2016.00020

[B24] Gahukar R. T. (2014). Factors affecting content and bioefficacy of neem (*Azadirachta indica* A. Juss.) phytochemicals used in agricultural pest control: a review. *Crop Prot.* 62 93–99. 10.1016/j.cropro.2014.04.014

[B25] Ghosh A. Chowdhury N. Chandra G. (2012). Plant extracts as potential mosquito larvicides. *Indian J. Med. Res.* 135 581–598. 22771587 PMC3401688

[B26] Giongo A. M. M. Vendramim J. D. Forim M. R. (2016). Evaluation of neem-based nanoformulations as alternative to control fall armyworm. *Ciênc. Agrotec.* 40 26–36. 10.1590/S1413-70542016000100002

[B27] Girish K. Shankara Bhat S. (2008). Neem–a green treasure. *Electron. J. Biol.* 4 102–111.

[B28] Gonzalez-Coloma A. Reina M. Diaz C. E. Fraga B. M. Santana-Meridas O. (2013). “Natural product-based biopesticides for insect control,” in *Reference Module in Chemistry, Molecular Sciences and Chemical Engineering* ed. Reedijk J. (Amsterdam: Elsevier).

[B29] Hasan F. Shafiq Ansari M. (2011). Toxic effects of neem-based insecticides on *Pieris brassicae* (Linn.). *Crop Prot.* 30 502–507. 10.1016/j.cropro.2010.11.029

[B30] Islam M. T. Omar D. Latif M. A. Morshed M. M. (2011). The integrated use of entomopathogenic fungus, *Beauveria bassiana* with botanical insecticide, neem against *Bemisia tabaci* on eggplant. *Afr. J. Microbiol. Res* 5 3409–3413.

[B31] Islam M. T. Omar D. B. (2012). Combined effect of *Beauveria bassiana* with neem on virulence of insect in case of two application approaches. *J. Anim. Plant Sci.* 22 77–82.

[B32] Isman M. B. (2006). Botanical Insecticides, Deterrents, and Repellents in Modern Agriculture and an Increasingly Regulated World. *Annu. Rev. Entomol.* 51 45–66. 10.1146/annurev.ento.51.110104.151146 16332203

[B33] Isman M. B. Grieneisen M. L. (2014). Botanical insecticide research: many publications, limited useful data. *Trends Plant Sci.* 19 140–145. 10.1016/j.tplants.2013.11.005 24332226

[B34] Isman M. B. Wan A. J. Passreiter C. M. (2001). Insecticidal activity of essential oils to the tobacco cutworm, *Spodoptera litura*. *Fitoterapia* 72 65–68. 10.1016/S0367-326X(00)00253-7 11163945

[B35] Joint Research Centre (2015). *Nano in Food and Agriculture: Regulations Require Collaboration to Ensure Safety – EU Science Hub – European Commission. EU Science Hub.* Available at: https://ec.europa.eu/jrc/en/news/nano-food-and-agriculture-regulations-require-collaboration-ensure-safety [accessed June 16, 2016].

[B36] Kah M. (2015). Nanopesticides and nanofertilizers: emerging contaminants or opportunities for risk mitigation? *Front. Chem.* 3:64 10.3389/fchem.2015.00064 PMC4644784 26636068

[B37] Kah M. Hofmann T. (2014). Nanopesticide research: current trends and future priorities. *Environ. Int.* 63 224–235. 10.1016/j.envint.2013.11.015 24333990

[B38] Kashyap P. L. Xiang X. Heiden P. (2015). Chitosan nanoparticle based delivery systems for sustainable agriculture. *Int. J. Biol. Macromol.* 77 36–51. 10.1016/j.ijbiomac.2015.02.039 25748851

[B39] Khot L. R. Sankaran S. Maja J. M. Ehsani R. Schuster E. W. (2012). Applications of nanomaterials in agricultural production and crop protection: a review. *Crop Prot.* 35 64–70. 10.1016/j.cropro.2012.01.007

[B40] Kogan M. (1998). Integrated pest management: historical perspectives and contemporary developments. *Annu. Rev. Entomol.* 43 243–270. 10.1146/annurev.ento.43.1.243 9444752

[B41] Kookana R. S. Boxall A. B. A. Reeves P. T. Ashauer R. Beulke S. Chaudhry Q. (2014). Nanopesticides: guiding principles for regulatory evaluation of environmental risks. *J. Agric. Food Chem.* 62 4227–4240. 10.1021/jf500232f 24754346

[B42] Kulkarni A. R. Soppimath K. S. Aminabhavi T. M. Dave A. M. Mehta M. H. (1999). Urea–formaldehyde crosslinked starch and guar gum matrices for encapsulation of natural liquid pesticide [*Azadirachta Indica* A. Juss.(neem) seed oil]: swelling and release kinetics. *J. Appl. Polym. Sci.* 73 2437–2446. 10.1002/(SICI)1097-4628(19990919)73:12<2437::AID-APP12>3.0.CO;2-7

[B43] Landis D. A. Wratten S. D. Gurr G. M. (2000). Habitat management to conserve natural enemies of arthropod pests in agriculture. *Annu. Rev. Entomol.* 45 175–201. 10.1146/annurev.ento.45.1.175 10761575

[B44] Li S. Y. Skinner A. C. Rideout T. Stone D. M. Crummey H. Holloway G. (2003). Lethal and sublethal effects of a neem-based insecticide on balsam fir sawfly (Hymenoptera: Diprionidae). *J. Econ. Entomol.* 96 35–42. 10.1093/jee/96.1.35 12650342

[B45] Lokanadhan S. Muthukrishnan P. Jeyaraman S. (2012). Neem products and their agricultural applications. *J. Biopestic.* 5 72–76. 10.1038/srep33484

[B46] Lucantoni L. Giusti F. Cristofaro M. Pasqualini L. Esposito F. Lupetti P. (2006). Effects of a neem extract on blood feeding, oviposition and oocyte ultrastructure in *Anopheles stephensi* Liston (Diptera: Culicidae). *Tissue Cell* 38 361–371. 10.1016/j.tice.2006.08.005 17097701

[B47] Mamoon-ur-Rashid M. Khattak M. K. Abdullah K. Hussain S. (2011). Toxic and residual activities of selected insecticides and neem oil against cotton mealybug, phenacoccus solenopsis tinsley (sternorrhyncha: pseudococcidae) under laboratory and field conditions. *Pak. Entomol.* 33 151–155.

[B48] Mancebo F. Hilje L. Mora G. A. Salazar R. (2002). Biological activity of two neem (*Azadirachta indica* A. Juss., Meliaceae) products on *Hypsipyla grandella* (Lepidoptera: Pyralidae) larvae. *Crop Prot.* 21 107–112. 10.1016/S0261-2194(01)00069-2

[B49] Mathur S. Kachhwaha S. (2015). Neem tree: amazing beauty component in skin and hair care. *Adv. Pharmacol. Toxicol.* 16 31–43.

[B50] Michereff-Filho M. Torres J. B. Andrade L. N. Nunes M. U. C. (2008). Effect of some biorational insecticides on Spodoptera eridania in organic cabbage. *Pest Manag. Sci.* 64 761–767. 10.1002/ps.1554 18300205

[B51] Miresmailli S. Isman M. B. (2014). Botanical insecticides inspired by plant–herbivore chemical interactions. *Trends Plant Sci.* 19 29–35. 10.1016/j.tplants.2013.10.002 24216132

[B52] Mohanty S. Patra A. Chhonkar P. (2008). Neem (*Azadirachta indica*) seed kernel powder retards urease and nitrification activities in different soils at contrasting moisture and temperature regimes. *Bioresour. Technol.* 99 894–899. 10.1016/j.biortech.2007.01.006 17360179

[B53] Montes-Molina J. A. Luna-Guido M. L. Espinoza-Paz N. Govaerts B. Gutierrez-Miceli F. A. Dendooven L. (2008). Are extracts of neem (*Azadirachta indica* A. Juss. (L.)) and *Gliricidia sepium* (Jacquin) an alternative to control pests on maize (*Zea mays* L.)? *Crop Prot.* 27 763–774. 10.1016/j.cropro.2007.11.002

[B54] Mordue (Luntz) A. J. Blackwell A. (1993). Azadirachtin: an update. *J. Insect Physiol.* 39 903–924. 10.4103/0973-7847.156337

[B55] Morgan E. D. (2009). Azadirachtin, a scientific gold mine. *Bioorg. Med. Chem.* 17 4096–4105. 10.1016/j.bmc.2008.11.081 19112026

[B56] Mossini S. A. G. Kemmelmeier C. (2005). A árvore Nim (*Azadirachta indica* A. Juss): múltiplos usos. *Acta Farm. Bonaer.* 24 139–148.

[B57] Mpumi N. Mtei K. Machunda R. Ndakidemi P. A. (2016). The toxicity, persistence and mode of actions of selected botanical pesticides in Africa against insect pests in common beans, *P. vulgaris*: a review. *Am. J. Plant Sci.* 7 138–151. 10.4236/ajps.2016.71015

[B58] Mulla M. S. Su T. (1999). Activity and biological effects of neem products against arthropods of medical and veterinary importance. *J. Am. Mosq. Control Assoc.* 15 133–152. 10412110

[B59] Musalia L. Anandan S. Sastry V. R. Agrawal D. (2000). Urea-treated neem (*Azadirachta indica* A. juss) seed kernel cake as a protein supplement for lambs. *Small Rumin. Res.* 35 107–116. 10.1016/S0921-4488(99)00085-1

[B60] Nair R. Varghese S. H. Nair B. G. Maekawa T. Yoshida Y. Kumar D. S. (2010). Nanoparticulate material delivery to plants. *Plant Sci.* 179 154–163. 10.1016/j.plantsci.2010.04.012

[B61] Ni K. Pacholski A. Kage H. (2014). Ammonia volatilization after application of urea to winter wheat over 3 years affected by novel urease and nitrification inhibitors. *Agric. Ecosyst. Environ.* 197 184–194. 10.1016/j.agee.2014.08.007

[B62] Nicoletti M. Petitto V. Gallo F. R. Multari G. Federici E. Palazzino G. (2012). The modern analytical determination of botanicals and similar novel natural products by the HPTLC fingerprint approach. *Stud. Nat. Prod. Chem.* 37 217–258.

[B63] Niu J.-Z. Hull-Sanders H. Zhang Y.-X. Lin J.-Z. Dou W. Wang J.-J. (2014). Biological control of arthropod pests in citrus orchards in China. *Biol. Control* 68 15–22. 10.1016/j.jip.2012.10.005

[B64] Norten E. Pütz J. (1999). *Neem: India’s Miraculous Healing Plant.* Rochester, VT: Inner Traditions/Bear & Co.

[B65] Ogbuewu I. P. Odoemenam V. U. Obikaonu H. O. Opara M. N. Emenalom O. O. Uchegbu M. C. (2011). The growing importance of neem (*Azadirachta indica* A. Juss) in agriculture, industry, medicine and environment: a review. *Res. J. Med. Plant* 5 230–245. 10.3923/rjmp.2011.230.245

[B66] Orr D. Lahiri S. (2014). “Chapter 23 – Biological control of insect pests in crops A2” in *Integrated Pest Management* ed. Abrol D. P. (San Diego, CA: Academic Press) 531–548.

[B67] Pal K. K. McSpadden Gardener B. (2006). Biological control of plant pathogens. *Plant Health Instr.* 2 1117–1142. 10.1094/PHI-A-2006-1117-02

[B68] Pant M. Dubey S. Patanjali P. K. (2016). “Recent advancements in bio-botanical pesticide formulation technology development,” in *Herbal Insecticides, Repellents and Biomedicines: Effectiveness and Commercialization* eds Veer V. Gopalakrishnan R. (New Delhi: Springer) 117–126.

[B69] Qiao J. Zou X. Lai D. Yan Y. Wang Q. Li W. (2014). Azadirachtin blocks the calcium channel and modulates the cholinergic miniature synaptic current in the central nervous system of *Drosophila*. *Pest Manag. Sci.* 70 1041–1047. 10.1002/ps.3644 24002996

[B70] Raguraman S. Kannan M. (2014). “Non-target effects of botanicals on beneficial arthropods with special reference to *Azadirachta indica*,” in *Advances in Plant Biopesticides* ed. Singh D. (New Delhi: Springer) 173–205.

[B71] Ram P. Vivek K. Kumar S. P. (2014). Nanotechnology in sustainable agriculture: present concerns and future aspects. *Afr. J. Biotechnol.* 13 705–713. 10.5897/AJBX2013.13554

[B72] Ramachandran S. Singh S. K. Larroche C. Soccol C. R. Pandey A. (2007). Oil cakes and their biotechnological applications – A review. *Bioresour. Technol.* 98 2000–2009. 10.1016/j.biortech.2006.08.002 17023161

[B73] Rehman J. U. Ali A. Khan I. A. (2014). Plant based products: use and development as repellents against mosquitoes: a review. *Fitoterapia* 95 65–74. 10.1016/j.fitote.2014.03.002 24631763

[B74] Riyajan S.-A. Sakdapipanich J. T. (2009). Encapsulated neem extract containing Azadiractin-A within hydrolyzed poly(vinyl acetate) for controlling its release and photodegradation stability. *Chem. Eng. J.* 152 591–597. 10.1016/j.cej.2009.05.017

[B75] Roychoudhury R. (2016). “Neem products,” in *Ecofriendly Pest Management for Food Security* ed. Omkar (Amsterdam: Elsevier) 545–562.

[B76] Sahayaraj K. Namasivayam S. K. R. Rathi J. M. (2011). Compatibility of entomopathogenic fungi with extracts of plants and commercial botanicals. *Afr. J. Biotechnol.* 10 933–938.

[B77] Scudeler E. L. dos Santos D. C. (2013). Effects of neem oil (*Azadirachta indica* A. Juss) on midgut cells of predatory larvae *Ceraeochrysa claveri* (Navás, 1911) (Neuroptera: Chrysopidae). *Micron* 44 125–132. 10.1016/j.micron.2012.05.009 22739123

[B78] Scudeler E. L. Garcia A. S. G. Padovani C. R. Santos D. C. (2013). Action of neem oil (*Azadirachta indica* A. Juss) on cocoon spinning in *Ceraeochrysa claveri* (Neuroptera: Chrysopidae). *Ecotoxicol. Environ. Saf.* 97 176–182. 10.1016/j.ecoenv.2013.08.008 23993219

[B79] Scudeler E. L. Padovani C. R. dos Santos D. C. (2014). Effects of neem oil (*Azadirachta indica* A. Juss) on the replacement of the midgut epithelium in the lacewing *Ceraeochrysa claveri* during larval-pupal metamorphosis. *Acta Histochem.* 116 771–780. 10.1016/j.acthis.2014.01.008 24560939

[B80] Seljåsen R. Meadow R. (2006). Effects of neem on oviposition and egg and larval development of *Mamestra brassicae* L: dose response, residual activity, repellent effect and systemic activity in cabbage plants. *Crop Prot.* 25 338–345. 10.1016/j.cropro.2005.05.007

[B81] Senthil-Nathan S. Choi M.-Y. Seo H.-Y. Paik C.-H. Kalaivani K. (2009). Toxicity and behavioral effect of 3β,2425-trihydroxycycloartane and beddomei lactone on the rice leaffolder *Cnaphalocrocis medinalis* (Guenée) (Lepidoptera: Pyralidae). *Ecotoxicol. Environ. Saf.* 72 1156–1162. 10.1016/j.ecoenv.2008.02.005 18397808

[B82] Senthil Nathan S. Kalaivani K. Sehoon K. Murugan K. (2006). The toxicity and behavioural effects of neem limonoids on *Cnaphalocrocis medinalis* (Guenée), the rice leaffolder. *Chemosphere* 62 1381–1387. 10.1016/j.chemosphere.2005.07.009 16194558

[B83] Servin A. D. White J. C. (2016). Nanotechnology in agriculture: next steps for understanding engineered nanoparticle exposure and risk. *Nanoimpact* 1 9–12. 10.1016/j.impact.2015.12.002

[B84] Seufert V. Ramankutty N. Foley J. A. (2012). Comparing the yields of organic and conventional agriculture. *Nature* 485 229–232. 10.1038/nature11069 22535250

[B85] Siegwart M. Graillot B. Blachere Lopez C. Besse S. Bardin M. Nicot P. C. (2015). Resistance to bio-insecticides or how to enhance their sustainability: a review. *Front. Plant Sci.* 6:381 10.3389/fpls.2015.00381 PMC4472983 26150820

[B86] Smith A. H. Capinera J. L. (2014). *Natural Enemies and Biological Control.* Available at: http://edis.ifas.ufl.edu/pdffiles/IN/IN12000.pdf

[B87] Srilatha B. (2011). Nanotechnology in agriculture. *J. Nanomed. Nanotechnol.* 2:123.

[B88] Sujarwo W. Keim A. P. Caneva G. Toniolo C. Nicoletti M. (2016). Ethnobotanical uses of neem (*Azadirachta indica* A. Juss.; Meliaceae) leaves in Bali (Indonesia) and the Indian subcontinent in relation with historical background and phytochemical properties. *J. Ethnopharmacol.* 189 186–193. 10.1016/j.jep.2016.05.014 27178630

[B89] Tangtrakulwanich K. Reddy G. V. P. (2014). “Development of insect resistance to plant biopesticides: an overview,” in *Advances in Plant Biopesticides* ed. Singh D. (New Delhi: Springer) 47–62.

[B90] Tavares W. S. Costa M. A. Cruz I. Silveira R. D. Serrão J. E. Zanuncio J. C. (2010). Selective effects of natural and synthetic insecticides on mortality of *Spodoptera frugiperda* (Lepidoptera: Noctuidae) and its predator *Eriopis connexa* (Coleoptera: Coccinellidae). *J. Environ. Sci. Health B* 45 557–561. 10.1080/03601234.2010.493493 20603748

[B91] Tunca H. Kilincer N. Ozkan C. (2012). Side-effects of some botanical insecticides and extracts on the parasitoid, Venturia canescens (Grav.) (Hymenoptera: Ichneumonidae). *Türk. Entomol. Derg.* 36 205–214.

[B92] Usman M. Inayatullah M. Sohail A. U. K. Shah S. F. (2012). Effect of egg parasitoid, Trichogramma chilonis, in combination with *Chrysoperla carnea* and neem seed extract against tomato fruitworm, *Helicoverpa Armigera*. *Sarhad J. Agric.* 28 1–5.

[B93] Venkateswarlu B. Pirat M. Kishore N. Rasul A. (2008). Mycorrhizal inoculation in neem (*Azadirachta indica*) enhances azadirachtin content in seed kernels. *World J. Microbiol. Biotechnol.* 24 1243–1247. 10.1007/s11274-007-9593-2

[B94] War A. R. Paulraj M. G. Ahmad T. Buhroo A. A. Hussain B. Ignacimuthu S. (2012). Mechanisms of plant defense against insect herbivores. *Plant Signal. Behav.* 7 1306–1320. 10.4161/psb.21663 22895106 PMC3493419

[B95] Weathersbee A. A. McKenzie C. L. (2005). Effect of a neem biopesticide on repellency, mortality, oviposition, and development of diaphorina citri (homoptera: psyllidae). *Fla. Entomol.* 88 401–407. 10.1653/0015-4040(2005)88[401:EOANBO]2.0.CO;2

[B96] Wilps H. Kirkilionis E. Muschenich K. (1992). The effects of neem oil and azadirachtin on mortality, flight activity, and energy metabolism of *Schistocerca gregaria* forskal—A comparison between laboratory and field locusts. *Comp. Biochem. Physiol. C Comp. Pharmacol.* 102 67–71. 10.1016/0742-8413(92)90045-9

[B97] Xu J. Fan Q.-J. Yin Z.-Q. Li X.-T. Du Y.-H. Jia R.-Y. (2010). The preparation of neem oil microemulsion (*Azadirachta indica*) and the comparison of acaricidal time between neem oil microemulsion and other formulations in vitro. *Vet. Parasitol.* 169 399–403. 10.1016/j.vetpar.2010.01.016 20304561

